# (−)-Oleocanthal as a Dual c-MET-COX2 Inhibitor for the Control of Lung Cancer

**DOI:** 10.3390/nu12061749

**Published:** 2020-06-11

**Authors:** Abu Bakar Siddique, Phillip C.S.R. Kilgore, Afsana Tajmim, Sitanshu S. Singh, Sharon A. Meyer, Seetharama D. Jois, Urska Cvek, Marjan Trutschl, Khalid A. El Sayed

**Affiliations:** 1 School of Basic Pharmaceutical and Toxicological Sciences, College of Pharmacy, University of Louisiana at Monroe, 1800 Bienville Drive, Monroe, LA 71201, USA; siddiqab@warhawks.ulm.edu (A.B.S.); tajmima@warhawks.ulm.edu (A.T.); singhss@warhawks.ulm.edu (S.S.S.); meyer@ulm.edu (S.A.M.); jois@ulm.edu (S.D.J.); 2 Department of Computer Science, Louisiana State University Shreveport, Shreveport, LA 71115, USA; pkilgore@lsus.edu (P.C.S.R.K.); urska.cvek@lsus.edu (U.C.); marjan.trutschl@lsus.edu (M.T.)

**Keywords:** (-)-Oleocanthal, c-MET, COX2, lung cancer, metastasis, microarray

## Abstract

Lung cancer (LC) represents the topmost mortality-causing cancer in the U.S. LC patients have overall poor survival rate with limited available treatment options. Dysregulation of the mesenchymal epithelial transition factor (c-MET) and cyclooxygenase 2 (COX2) initiates aggressive LC profile in a subset of patients. The Mediterranean extra-virgin olive oil (EVOO)-rich diet already documented to reduce multiple malignancies incidence. (-)-Oleocanthal (OC) is a naturally occurring phenolic secoiridoid exclusively occurring in EVOO and showed documented anti-breast and other cancer activities via targeting c-MET. This study shows the novel ability of OC to suppress LC progression and metastasis through dual targeting of c-MET and COX-2. Western blot analysis and COX enzymatic assay showed significant reduction in the total and activated c-MET levels and inhibition of COX1/2 activity in the lung adenocarcinoma cells A549 and NCI-H322M, in vitro. In addition, OC treatment caused a dose-dependent inhibition of the HGF-induced LC cells migration. Daily oral treatment with 10 mg/kg OC for 8 weeks significantly suppressed the LC A549-Luc progression and prevented metastasis to brain and other organs in a nude mouse tail vein injection model. Further, microarray data of OC-treated lung tumors showed a distinct gene signature that confirmed the dual targeting of c-MET and COX2. Thus, the EVOO-based OC is an effective lead with translational potential for use as a prospective nutraceutical to control LC progression and metastasis.

## 1. Introduction

The American Cancer Society estimated 1.8 million new cancer cases to be diagnosed this year, of which 228,820 (116,300 men and 112,520 women) will be new lung cancer (LC) cases [1]. The 2020 death toll expected in the U.S. includes 135,720 patients, of which 72,500 men and 63,220 women. This mortality profile ultimately represents the topmost of any cancer type [1,2]. About 85% of lung tumors are non-small cell lung cancer (NSCLC) and ~15% are small cell lung cancer (SCLC) [3,4,5,6]. There are also other rare LC types like large cell carcinoma, adenosquamous cell carcinoma, and sarcomatoid carcinoma. NSCLC is subdivided to squamous cell lung carcinoma (SCC) and lung adenocarcinoma (LUAD) [3]. The LUAD oncogenic drivers and their frequency include—Kirsten rat sarcoma viral oncogene homolog oncogene (KRAS, mutation, ~27%), epidermal growth factor receptor (EGFR, mutation, 25%), fibroblast growth factor receptor 1 (FGFR1, amplification, 20%), c-MET (amplification, 10–12%), HER2 (amplification, 9%), anaplastic lymphoma kinase (ALK, rearrangement, 6%), v-Raf murine sarcoma viral oncogene homolog B (BRAF, mutation, 2%), receptor tyrosine kinase (RET, rearrangement, 1%), neurotrophic tyrosine receptor kinase 1 (NTRK1, rearrangement, 1%), ROS proto-oncogene 1 receptor tyrosine kinase (ROS1, rearrangement, 1%) [3,5,6]. The SCC oncogenic drivers include phosphatase and tensin homolog (PTEN), phosphatidylinositol-4,5-bisphosphate 3-kinase catalytic subunit alpha (PIK3CA) and FGFR1 [7,8,9,10]. 

MET or hepatocyte growth factor (HGF) receptor signaling pathway mediates non-pathological functions including wound healing and hepatic regeneration, with pivotal roles in embryonic, neuronal and muscle development [11]. However, dysregulation of hepatocyte growth factor receptor (c-MET) significantly correlates with aggressive proliferation, invasive and pathological motility profiles in several malignancies [11,12], particularly NSCLC, gastrointestinal (GI) cancer and hepatocellular carcinoma (HCC) [13,14,15,16]. Aberrant MET signaling can occur through several mechanisms including HGF overexpression, MET overexpression, amplification, mutation, fusion or rearrangement or aberrations in downstream signaling or regulatory components. MET signaling activates MAPK, ERK and PI3K/AKT pathways, bypassing the requirement for EGFR signaling [7,8,17,18,19]. Currently, three different approaches are available to target c-MET kinase activity including—(i) antibodies, which prevent the extracellular binding of c-MET with HGF, (ii) small-molecules, which selectively prevent the phosphorylation of specific tyrosine residues in the kinase domain and (iii) blocking the c-MET kinase-dependent signaling through relevant signal transducers or downstream signaling components [20]. Responses to MET tyrosine kinase inhibitors have been documented in clinical trials in patients with MET-amplified or MET-overexpressing NSCLC. Crizotinib is a MET/ALK/ROS1 tyrosine kinase inhibitor (TKI) approved for ALK-driven LC [9,21,22]. LC patients with ALK rearrangements displayed ~61% objective response rate to crizotinib in Phase I trials [9]. c-MET amplification also correlates with LC escape from the anticancer effects of several targeted therapies, including 5–15% of patients using first-generation EGFR TKIs [7,8,17,18]. Thus, MET inhibitors combined with EGFR TKIs have been successfully evaluated in several clinical trials [20]. Eventually, c-MET has become an attractive target for LC targeted control. Although several agents targeting c-MET have been examined in clinical trials but the results range from relatively high response rates to prominent failure, which clearly justify the need for more comprehensive studies to understand the role of c-MET in cancer pathogenesis and the crucial need for the discovery of new c-MET inhibitors with potential novel binding modes and cost-effectiveness. 

On the other hand, COX enzymes generally played important roles in the inflammation cascade but several studies revealed significant roles of cyclooxygenases, especially COX2, in breast [23], lung [24], colon [25], stomach [26], and head and neck squamous cell carcinoma [27]. In NSCLC, COX2 is overexpressed in most adenocarcinomas and squamous cell carcinomas [28]. Elevated tumor COX2 and prostaglandin E2 (PGE2) levels have been implicated in over-activated angiogenesis, tumor invasion, resistance to apoptosis, and suppression of antitumor immunity [28]. Preclinical animal model studies have already documented the significant tumor reduction upon nonspecific or specific inhibition of COX2 activity. Celecoxib, a selective COX2 inhibitor has been evaluated in combination with chemotherapy for the management of metastatic NSCLC in patients who have failed prior chemotherapy [29]. Interestingly, HGF proved to increase the level of COX2 expression up to 3-fold over basal levels [30]. This induction of COX2 occurred through both the extracellular signal-regulated kinase (ERK) 1/2 and p38 pathways [30]. Additional studies showed that co-targeting c-MET and COX2 led to a significant decrease in the number of lung tumors per animal after 13-week treatments with crizotinib, celecoxib or their combination, versus the placebo control [31]. Several preclinical and clinical studies have demonstrated that COX2 inhibitors are effective for the control of NSCLC [32]. Two recent phase III clinical trials used COX2 inhibitors in combination with platinum-based chemotherapy failed to demonstrate a survival benefit due to “unselected” advanced NSCLC patients in a randomized phase III trial [32]. However, the overall COX2 inhibitors-treated group had improved response rate with chemotherapy, yet had no effect on survival indices. 

There is mounting epidemiological and clinical evidences that validated the extra-virgin olive oil (EVOO)-rich Mediterranean diet ability to reduce the incidence of multiple cancers [33,34,35,36]. EVOO proved to reduce the oxidative stress and inflammation and exert acute anti-platelet aggregation activity in human clinical trials [21,22,37]. EVOO polyphenols are minor secondary metabolites with diverse phenolic structural classes [38]. Among these, the monophenol secoiridoid *S*-(-)-oleocanthal (OC) attracted the topmost scientific attention due to its exceptional biological activities in different therapeutic directions even though it makes only around 10% of the EVOO total phenolics content [38,39]. OC exerted its anti-inflammatory effect by concurrently inhibiting COX1/2, which suppressed the biosynthesis of prostaglandins and thromboxanes from arachidonic acid and 5-LOX, translating to equipotent anti-inflammatory activity to the common non-steroidal anti-inflammatory drug (NSAID) ibuprofen [40]. Earlier, OC effectively suppressed the progression of the triple negative breast cancer (TNBC) via targeting the c-MET receptor tyrosine kinase (RTK) [41,42,43,44]. OC inhibited in vitro and in vivo progression of several BC cell lines, including TNBC and the luminal B ER^+^/HER2^+^ BC phenotypes and synergized with the selective estrogen receptor modulator tamoxifen [41,42,43,44,45]. In addition, OC exhibited potent synergistic activity with the dual EGFR-HER2 RTKI lapatinib both in vitro and in vivo [46]. Recently, for the first time, we showed that daily oral 10 mg/kg OC prevented the locoregional recurrence of the ER^+^/HER2^+^ BC in a nude mouse orthotopic xenograft model after primary tumor surgical excision and after neoadjuvant lapatinib regimen completion and in adjuvant treatment mode [47]. Novel OC formulations developed and tested and proven to effectively suppress the locoregional recurrence of the TNBC MDA-MB-231 and HER2-overexpressing luminal B BT-474 BC cells in nude mouse orthotopic xenograft models after the primary tumor surgical excision [48,49]. OC has already shown promising in vitro and in vivo anticancer effects against melanoma [50], breast and prostate cancers [41,42,43,44,51], hepatocellular carcinoma [52,53], colon cancer [52,54], multiple myeloma [55], and leukemia [56]. OC showed high degree of selectivity to cancerous cells and exhibited modest or no cytotoxic effects against the non-tumorigenic cell lines, including the human adult dermal fibroblast HDFa cells [57], human mammary epithelial MCF10A cells [42], human liver LO2 cells [53], murine macrophages J774A.1 cells [55], human fibroblast BJ cells [58], rat fibroblast 3Y1 cells [58], human lung fibroblast IMR90 cells [58] and isolated primary human hepatocytes [52]. Recent single dose acute safety study further suggested the potential safety profile of OC single oral therapeutic dose (10 mg/kg) in Swiss albino mouse model over 14 days of administration [59]. OC inhibited the metastasis of melanoma and hepatocellular carcinoma to lung in mouse tail vein models after iv injection of A431 melanoma and HCCLM3 hepatocellular cancer cells, respectively [52,53]. However, there is no studies yet explored the direct anti-LC activity of OC. Thus, this study reports for the first time the OC effective suppression of LC progression, migration, and metastasis, in vitro and in vivo. This anti-LC activity proved to be via the dual targeting of c-MET and COX2, validating OC as an appealing nutraceutical lead intervention to control lung malignancies. 

## 2. Materials and Methods

### 2.1. Chemicals, Reagents and Antibodies

All reagents purchased from VWR International (Suwanee, GA, USA), unless otherwise stated. All primary, secondary antibodies were purchased from Cell Signaling Technology (Beverly, MA, USA), unless otherwise stated. Hepatocyte growth factor (HGF) purchased from PeproTech Inc. (Rocky Hill, NJ, USA).

### 2.2. Extraction, Purification and Analysis of (-)-Oleocanthal from Extra-Virgin Olive Oil

OC was extracted from EVOO (The Governor, batch #5-214000-242017). Separation was performed using the previously reported liquid–liquid extraction technology, extracting EVOO with water, resin entrapment, followed by ^1^H NMR-guided size exclusion chromatography on Sephadex LH20, using isocratic CH_2_Cl_2_ elution. Pure OC sample was stored at −20 °C in amber glass vials under N_2_ gas until used for animal dosing [45]. OC purification and analysis methods extensively described earlier in Siddique, et al. 2019 [45].

### 2.3. Cell Lines and Culture Conditions

The human NSCLC cell line A549-Luc was obtained from Perkin Elmer Inc. (Santa Clara, CA, USA). The A549 and the human lung endothelial cells HMVEC were obtained from the American Type Culture Collection (ATCC, Manassas, VA, USA). The human LC cell line NCI-H322M was obtained from Charles River Laboratories (Frederick, MD, USA). The A549, A549-Luc and NCI-H322M cells were cultured in Roswell Park Memorial Institute (RPMI-1640)/Dulbecco’s Modified Eagle’s medium (DMEM) media supplemented with 10% fetal bovine serum (FBS), penicillin G (100 U/mL) and streptomycin (100 ng/mL). All cells were maintained in a humidified incubator at 37 °C with 5% CO_2_. For sub-culturing, cells were washed with Ca^2+^- and Mg^2+^-free phosphate-buffered saline (PBS) and incubated in 0.05% trypsin containing 0.02% ethylenediamine-tetraacetic acid (EDTA) in PBS for 5–15 min at 37 °C.

### 2.4. Experimental Treatments

OC dissolved in sterile dimethyl sulfoxide (DMSO) to provide a 10 mM stock solution. These stock solutions used to prepare various treatment concentrations. The final concentration of DMSO maintained the same in all treatment groups within a given experiment and never exceeded 0.1% in sterile PBS [45].

### 2.5. Cell Viability Assay

Cells were seeded into 96-well plates at a density of 1 × 10^4^ cells/well (6 replicates/group) in 10% FBS RPMI-1640 media and left to attach overnight. Next day, cells were divided into different treatment groups and exposed to respective control or experimental treatments with various concentrations of OC or vehicle control for 48 h in media with or without 40 ng/mL of HGF as a mitogen. At the end of treatment duration, the viable cell number was quantified using 3-(4,5-dimethylthiazolyl-2)-2,5-diphenyltetrazolium bromide (MTT) assay [42,43,45,46]. MTT was added to each well at a final concentration of 1.0 mg/mL. After 4 h incubation at 37 °C, media was removed and formazan crystals were dissolved in DMSO (100 µL/well). Optical density was measured at 570 nm on a microplate reader (BioTek, Winooski, VT, USA). The number of cells/well was calculated against a standard curve prepared by plating various numbers of cells at the beginning of each experiment.

### 2.6. Western Blot Analysis

LC cells were initially plated at 1 × 10^6^ cells/10 cm culture plates in Roswell Park Memorial Institute (RPMI)-1640 media supplemented with 10% FBS and allowed to adhere overnight. Cells were then washed with PBS and treated with the respective control or treatment media containing various concentrations of OC for 48 h with or without containing 40 ng/mL HGF as mitogen. Cells were then harvested and washed twice with cold PBS, resuspended and lysed in Radioimmuno-precipitation assay (RIPA) buffer (Qiagen Sciences Inc., Valencia, CA, USA) at 4 °C for 30 min. Lysates were centrifuged for 10 min at 14,000× g and supernatants were stored at −80 °C as whole cell extracts. Lung tumor tissue samples were collected and immediately stored at −80 °C until protein extraction. Tumor tissues were homogenized in RIPA buffer using an electric homogenizer. Protein concentration was determined by the Pierce BCA Protein Assay (Thermo Fisher Scientific Inc., Rockford, IL, USA). Proteins were separated on 10% sodium dodecyl sulfate polyacrylamide gel electrophoresis (SDS-PAGE) gels and transferred to polyvinylidene difluoride membranes. Membranes blocked with 2% bovine serum albumin (BSA) and incubated with the indicated primary antibodies. Corresponding horseradish peroxidase-conjugated secondary antibodies were used against each primary antibody. Proteins were detected using ChemiDoc XRS chemiluminescent gel imaging system and analyzed using Image Lab software (BioRAD, Hercules, CA, USA) [42,43,45,46,47]. Visualization of β-tubulin was used to ensure equal sample loading in each lane. Experiments were repeated three times and representative image presented in each figure.

### 2.7. Wound-Healing Assay

The in vitro wound-healing assay was used to assess directional two-dimensional cell motility. A549 and NCI-H322M cells were plated in sterile flat-bottom 24-well plates (3 replicates/group) and allowed to form a sub-confluent cell monolayer per well overnight. Wounds were then scratched in each cell monolayer using a 200 µL-sterile pipette tip. Media was removed and cells were washed twice with PBS and once with fresh serum-free media to remove floating cells. Cells were then incubated in culture media containing OC with different doses in 0.5% serum containing media with or without 40 ng/mL HGF as the mitogen. Cells were incubated for a 24 h or until wound was closes in vehicle control wells. Media was removed and cells were washed with pre-cooled PBS, fixed with methanol previously cooled to −20 °C and stained with Giemsa. Wound healing was visualized at 0 time and 24 h or until close the wound of vehicle control by Nikon ECLIPSE TE200-U microscope (Nikon Instruments Inc., Melville, NY, USA). Digital images were captured using Nikon NIS Elements software (Nikon Instruments Inc., Melville, NY, USA). The distance traveled by the cells was determined by measuring the wound width at time 24 h or ending the experiments hours and subtracting it from the wound width at the start of treatment (zero time). The values obtained were then expressed as % migration, setting the gap width at the t0 as 100%. Each experiment was conducted in triplicate and the distance migrated was calculated in three or more randomly selected fields per treatment group [42].

### 2.8. COX1 and COX2 Enzymatic Assay

COX1 and COX2 inhibition assay used the COX Activity Assay kit (cat # 760151, Cayman Chemicals, Ann Arbor, MI, USA) to investigate the OC treatments effect on the enzymatic activity of COX enzymes versus the standard COX inhibitors DuP-697 (Cayman Chemicals, cat # 70645) and SC-560 (Cayman Chemicals, cat # 70340). DuP-697 is a selective COX2 diaryl heterocyclic inhibitor while the SC-560 is a selective COX-1 inhibitor. Cells lysate samples were processed and each sample was assessed run as described by Cayman Chemicals. The colorimetric COX assay was measured by monitoring the appearance of colorimetric oxidized *N,N,N*′,*N*′-tetramethyl-*p*-phenylenediamine (TMPD) at 590 nm, which was translated to COX activity as per the manufacturer’s protocol.

### 2.9. In Vivo Studies

#### 2.9.1. Animals

Athymic nude mice (Foxn1^nu^/Foxn^1+^, female, 4–5 weeks) were purchased from Envigo (Indianapolis, IN, USA). The animals were acclimated to the animal housing facility and maintained under clean room conditions in sterile filter-top cages with Alpha-Dri bedding and housed on high efficiency particulate air-filtered ventilated racks at a temperature of 18–25 °C, with a relative humidity of 55–65% and a 12 h light/dark cycle, for at least one week before the study. The mice had free access to drinking water and pelleted rodent chow (no. 7012, Envigo/Teklad, Madison, WI, USA). Animals were housed in group cages, each *n* = 4 animals/experimental group. All animal experiments were approved by the Institutional Animal Care and Use Committee (IACUC), University of Louisiana at Monroe and were conducted in strict accordance with good animal practice as defined by NIH guidelines (Protocol# 18OCT-KES-02).

#### 2.9.2. LC Nude Mouse Tail Vein Injection Model

After acclimatization of animals to the local environment for a week, nearly 5 × 10^6^ Luciferase- transfected A549-Red-FLuc Bioware^®^ Brite Cell Line (Perkin Elmer, Santa Clara, CA, USA) in 100 µL sterile PBS were injected intravenously into each mouse tail vein. Bioluminescence was measured by imaging 2% isoflurane anesthetized mice using an IVIS Lumina series III (Perkin Elmer) imaging system after intraperitonially (ip) injecting with D-luciferin (XenoLight D-luciferin K^+^ salt bioluminescent Substrate, PerkinElmer) at a dose of 150 mg/kg per animal in sterile PBS. The photons emitted from luciferase-expressing cells within the animal body and transmitted through the tissue were quantified using the Living Image software program (PerkinElmer). Images representing light intensity (blue least intense and red most intense) was generated and quantified as photons/second. On the same day of tumor inoculation, mice were randomized to two groups, *n* = 4 each (i) vehicle control and (ii) OC 10 mg/kg daily orally treated group after confirming each mouse having injected cells properly. The mice were treated for 8 weeks. OC was administered at a dose of 10 mg/kg orally everyday while the vehicle control group received saline water. Animals were imaged and bioluminescence were recorded once a week to monitor the progression of lung cancer. The animal’s health status was monitored routinely for weight loss or any signs of altered motor ability while in their cages. At the end of the study, mice were sacrificed according to the approved IACUC protocol. Lung tumors, along with other organs (brain, heart, liver, kidney and pancreas) from all animals were harvested and embedded in paraffin for subsequent analysis. All data are presented as the mean ±SD. Statistical differences were evaluated by student t-test analysis of data from different two groups and the criterion for statistical significance was *p* < 0.05.

### 2.10. Hematoxylin and Eosin Y (H&E) Staining

Lung tissues were freshly collected and immediately fixed in 10% neutral buffered formalin for 48 h. The tissues were further transferred to 70% ethanol, processed and embedded in paraffin. All the sectioning and H&E staining has been done at the AML Laboratories (Jacksonville, FL, USA). Briefly, paraffin-embedded tissues were sliced into 5 µm sections and mounted on positively charged slides, dewaxed with xylene, rinsed with alcohol, rehydrated by water and finally, the tissue slides were stained with H&E. Tissues were then dehydrated through ethanol to xylene and coverslipped with Permount [59].

### 2.11. Transcription Microarray

Total RNA isolated using the Triozol-PhaseLock Gel protocol. Quality control of the isolated RNA was performed using Agilent TapeStation 4200 RNA ScreenTape assay kit (Agilent, 5067-5576) to determine the RNA integrity number (RIN) and concentration of the RNA. QC-passed RNA samples further processed for target labeling using the Clariom S Human array/WT plus assay kit (Life Technologies, Cat# 902926). An RNA ScreenTape assay of the biotin-labeled targets performed prior sample hybridizations. QC-passed samples hybridized to the Clariom S, Human array using GeneChip system. All array data sets uploaded onto the microarray data management system (MDMS) for storage. All array experiments were performed at the University of Kansas Medical Center Genomics Core Facility (Kansas City, KS, USA).

### 2.12. Transcription Microarray Data Analysis 

The data was converted to fold-change values using the Affymetrix’s Transcriptome Analysis Console, applying the Clariom S human library. Values collected with an absolute fold-change of at least 2.0 and higher. Each probe’s corresponding GO biological process terms was appended to the data and retained only those probes, which had a term containing the phrases “cell proliferation,” “migration” and “recurrence.” This resulted in 715 selected genes. A standard core analysis was then performed on the preprocessed data. The Ingenuity Knowledge database was utilized as a reference and included both direct and indirect relationships. About 25 interaction networks of up to 35 molecules each, including endogenous chemicals, selecting all available node types and pulling from all available data sources was generated. We then compared data against experimentally observed sources only, which included human, mouse, rat, as well as uncategorized sources. All available cell lines were used and all mutations were allowed. To calculate the genes with >10% expression, 10% of the control expression c for each gene (ɛ) was calculated. The gene was retained if its treatment expression t was such that t > c + ɛ or t < c − ɛ. To obtain a general picture of IPA-predicted pathways, functions and molecular networks induced, data exported and the top and bottom 10 instances of each set were selected.

### 2.13. Statistics

Values are expressed as mean ± standard deviation (SD) and analyzed using the statistical package for GraphPad Prism software version 8 using student t-test or differences among various treatment groups were determined by one-way analysis of variance (ANOVA) followed by Tukey’s test; *p* < 0.05 was considered statistically significant.

## 3. Results

### 3.1. HGF Enhances LC Cells Viability

**Figure 1:**
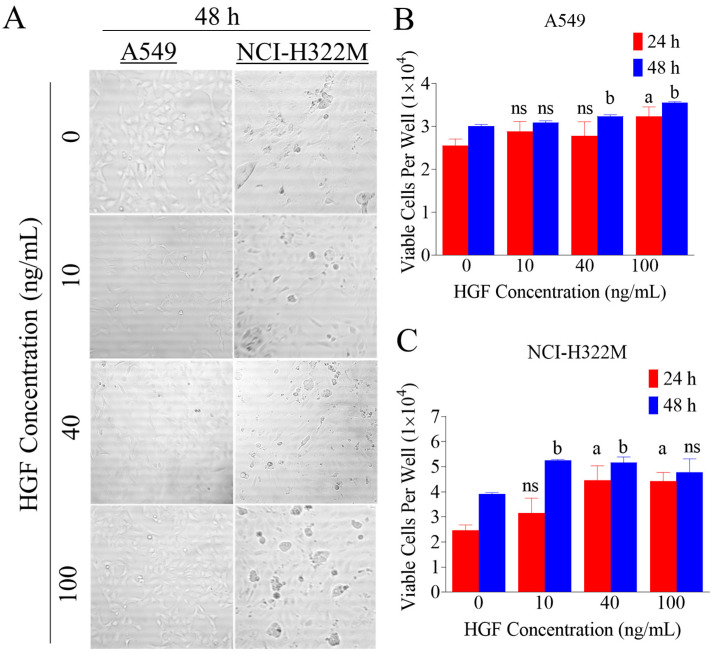
Effect of increasing hepatocyte growth factor (HGF) treatments on lung cancer (LC) cells proliferation. (**A**) Representative image of A549 and NCI-H322M LC cells viability assay in 96 well plate with increasing HGF concentrations after 48 h. (**B**,**C**) HGF stimulated the human LC cells (A549 and NCI-H322M) proliferation in a dose-dependent manner, reaching a maximum effect at 40 ng/mL over 48 h culture period. Cells were plated at a density of 1 × 10^4^ cells/well in 96-well plates and maintained in 10% FBS supplemented media and allowed to adhere overnight. The next day, cells were washed with PBS, divided into different HGF treatment groups. Viable cells count was determined by MTT assay after 48 h. Vertical bars indicate the mean cell count ±SD in each treatment group. ^a^ *p* < 0.05 significantly different compared to vehicle-treated controls at 24 h and ^b^ *p* < 0.05 significantly different, compared to vehicle-treated controls at 48 h, ns: statically not significant.

The activation of c-MET by HGF is well-known to induce cells viability and proliferation in many kinds of cancer cells. Consequently, dose-response effect of increasing HGF treatments was studied on the viability of A549 and NCI-H322M LC cells (Figure 1). Proliferation assay was performed in both LC cells treated with 0, 10, 40 and 100 ng/mL HGF (Figure 1A). The assay results were measured after 24 and 48 h of treatments. HGF caused an increase in LC cells proliferation (Figure 1B,C), reaching the maximum effect at 40 ng/mL, which was comparable to 100 ng/mL in all tested LC cell lines (Figure 1B,C).

### 3.2. Effects of OC Treatments on HGF-Induced LC Cells Viability

**Figure 2:**
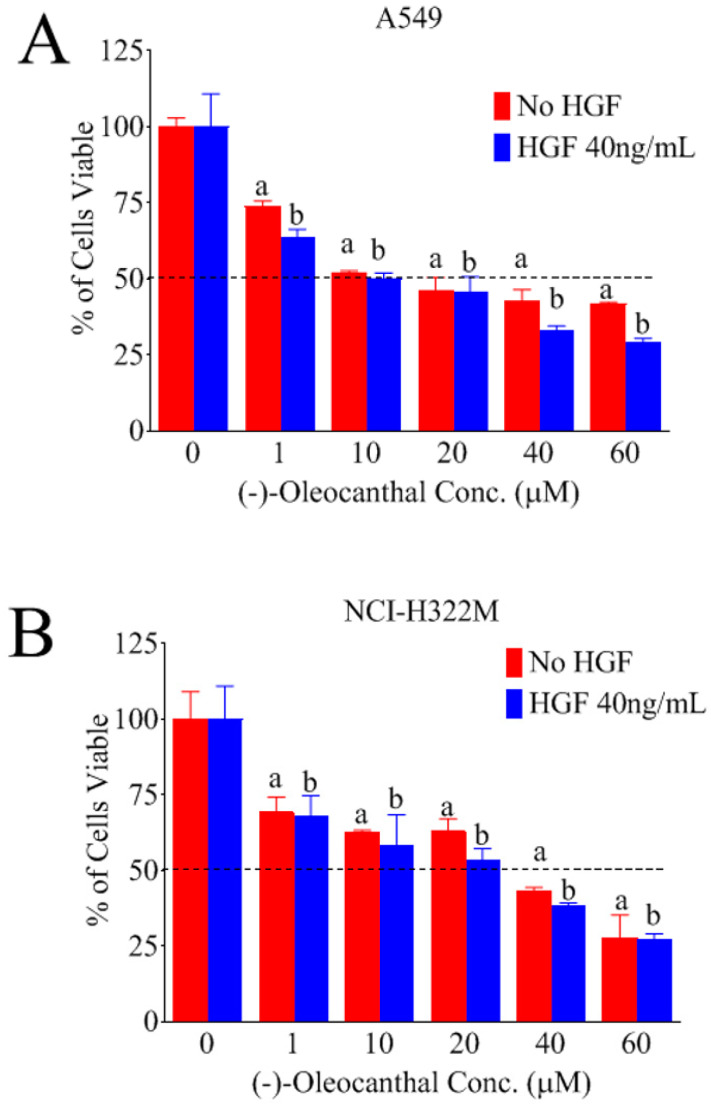

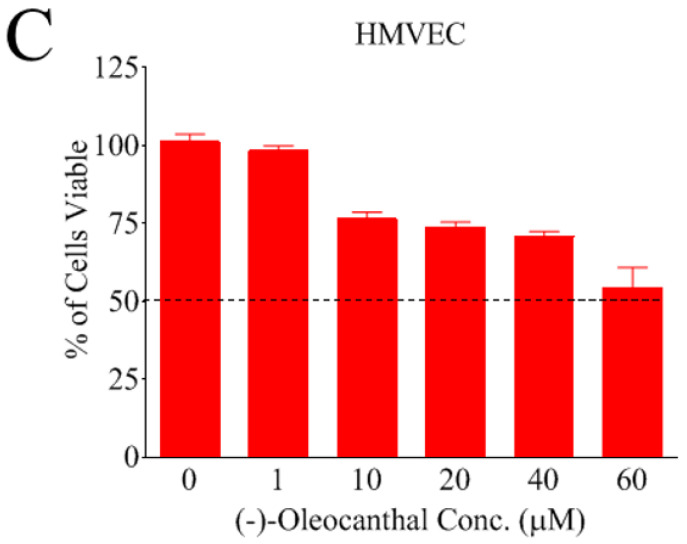
(-)-Oleocanthal selectively inhibited the viability of A549 and NCI-H322M LC cell lines and minimally affected the non-tumorigenic human microvascular endothelial cells viability. Effects of Oleocanthal (OC) treatment against the growth of A549 (**A**) and NCI-H322M (**B**) LC cells in the presence or absence of 40 ng/mL mitogenic HGF over 48 h treatment period. (**C**) Effects of OC treatment on the viability of the non-tumorigenic human microvascular endothelial cells (HMVEC) over 48 h treatment period. Cells were plated at a density of 1 × 10^4^ cells per well in 96-well plates and maintained in media supplemented with 10% fetal bovine serum (FBS) and allowed to adhere overnight. The next day, cells were washed with phosphate-buffered saline (PBS), divided into HGF or no HGF treatment groups. Viable cells count was determined by MTT assay at 48 h. Vertical bars indicate the mean cell count ±SD in each treatment group. ^a^ *p* < 0.05 significantly different compared to vehicle-treated control without HGF and ^b^ *p* < 0.05 significantly different compared to vehicle-treated control with HGF 40 ng/mL over 48 h.

The effects of various OC doses on the HGF-mediated growth of A549 and NCI-H322M LC cells after 48 h culture periods was investigated (Figure 2). HGF 40 ng/mL was used in growth studies based on previous experiment. OC treatment caused a dose-dependent suppression of the HGF-induced growth of the LC cells A549 (Figure 2A) and NCI-H322M (Figure 2B) over 48 h treatment period. However, larger OC concentrations were required to significantly abolish the A549 and NCI-H322M cells viability in HGF-free media over 48 h (Figure 2A,B). The IC_50_ values for OC treatment in HGF-supplemented media were 10.1 and 26.3 µM against A549 and NCI-H322M LC cells, respectively. However, the IC_50_ values for OC treatment in HGF-free media were 16.5 and 31.3 µM in A549 and NCI-H322M LC cells, respectively. These results indicate that OC is more effective in inhibiting the A549 and NCI-H322M LC cells growth in presence of HGF compared to HGF-free and vehicle-treated control group.

### 3.3. Effects of OC Treatments on Non-tumorigenic Human Microvascular Endothelial Cells Viability

Study of the effects of OC treatment on the growth and viability of the immortalized non-tumorigenic human microvascular endothelial cells (HMVEC) over 48 h culture periods indicated that up to 40 µM OC had little effect on HMVEC cells viability versus vehicle-treated control groups (Figure 2C). In contrast, OC 60 µM treatment started to show significant cell growth and viability inhibition over 48 h. These results suggest the optimal selectivity of OC, nearly 2-fold, toward LC versus the non-tumorigenic cells.

### 3.4. Effects of OC Treatments on HGF-Induced c-MET Expression and Phosphorylation

**Figure 3:**
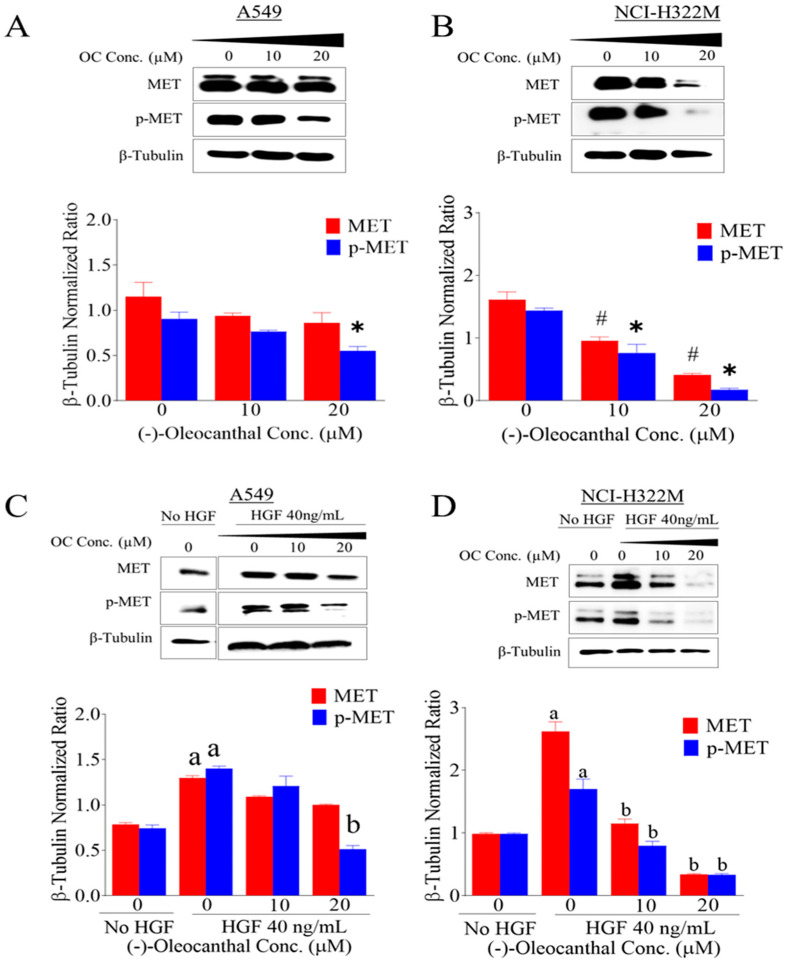
The in vitro effects of OC treatments on the total and activated c-MET levels in human LC cells. Western blot analysis for OC treatment effects without HGF (**A**) A549 and (**B**) NCI-H322M cells and with HGF (40 ng/mL) (**C**) A549 and (**D**) NCI-H322M cells on the total and activated c-MET levels. Cells were plated at 1 × 10^6^ cells/100 mm culture plates in RPMI-1640 media supplemented with 10% FBS and allowed to adhere overnight. Cells were then washed twice with PBS and starved in both control and treatment media containing either vehicle control or OC treatments. In case of HGF, cells were separately treated with either vehicle control or OC treatments containing 40 ng/mL HGF for 48 h. Scanning densitometry was obtained for each blot, carried out in triplicate and the integrated optical density of each band was normalized with the corresponding density found for β-tubulin in the same blot. The bottom panel vertical bar graphs indicate the normalized integrated bands of total and activated c-MET optical density visualized in each lane ±SD. ^*^ and # *p* < 0.05 significant difference between vehicle control and OC treatment. ^a^ *p* < 0.05 significant difference between cells cultured without HGF treatment and with 40 ng/mL HGF over 48 h. ^b^ *p* < 0.05 significant difference between OC treatments and vehicle-treated controls in presence of 40 ng/mL HGF over 48 h.

The human LC cell lines A549 and NCI-H322M were used to assess the effect of OC treatments on the HGF-induced c-MET activation (phosphorylation) by Western blotting (Figure 3). OC treatment without HGF, significantly inhibited the activation of c-MET at higher dose (Figure 3A,B) in both cell lines. Phospho-c-MET refers to the phosphorylation of the kinase domain at Y1234/1235. Interestingly, results showed that OC treatments caused a dose-dependent inhibition of c-MET phosphorylation in presence of mitogenic HGF treatment (40 ng/mL) in both investigated LC cell lines. However, higher OC treatment doses concomitantly and significantly suppressed the total and activated levels of c-MET in both LC cell lines (Figure 3C,D). The NCI-H322M cells were more sensitive to OC treatments versus the wild-type EGFR NSCLC cells A549 by showing marked decrease of the total and activated levels of c-MET as suggested by Western blotting (Figure 3C,D).

### 3.5. Effects of OC Treatments on The COX1/2 Activity in HGF-Treated A549 LC Cells

**Figure 4:**
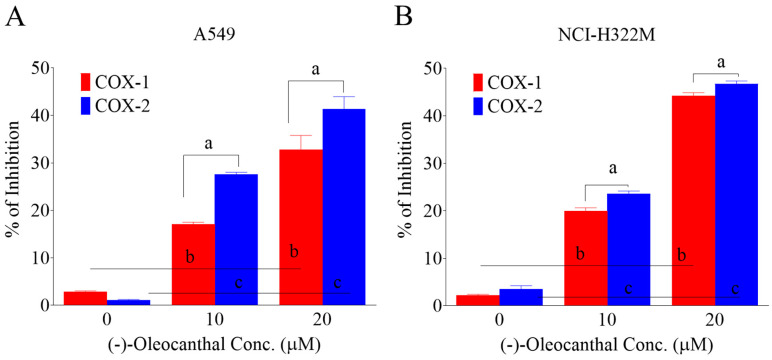
Suppressive effects of OC treatments on the activity of COX1/2 levels in lung can cencer (**A**) A549 cells and (**B**) NCI-H322M cells. Cells were plated at 1 × 10^6^ cells/100 mm culture plates in RPMI-1640 media supplemented with 10% FBS and allowed to adhere overnight. Cells were then washed twice with PBS and starved in control or OC treatments in media containing 40 ng/mL HGF for 48 h. Cells were then homogenized and lysed and the assay experiments followed the manufacturer protocol (Cayman Chemicals). SC-560 used as a COX-1 standard inhibitor while DuP-697 used as a standard COX-2 inhibitor according to the manufacturer protocol. Vertical bars in the graph indicate the % inhibition of COX1/2 ± SD. ^a^ *p* < 0.05 significant variation of OC treatment effects on COX1 versus COX2 activity at the same doses. ^b^ *p* < 0.05 significant variation between vehicle control and OC treatment effects on COX1 inhibition. ^c^ *p* < 0.05 significant variation between vehicle control and OC treatment effects on COX2 inhibition.

OC treatments dose-dependently suppressed the activity of COX1/2 levels in A549 and NCI-H322M LC cells as indicated by enzymatic assay results (Figure 4A,B). In A549 cells OC treatment doses of 10.0 and 20.0 µM resulted in 17.1% and 32.8% COX1 activity reductions, respectively. Similarly, OC 10.0 and 20.0 µM treatments caused 27.6% and 41.4% COX2 activity reductions, respectively (Figure 4A). In NCI-H322M cells OC treatment doses of 10.0 and 20.0 µM resulted in 20% and 44.3% COX1 activity reductions, respectively. Similarly, OC 10.0 and 20.0 µM treatments caused 23.6% and 46.7% COX2 activity reductions, respectively (Figure 4B). These results indicate the OC ability to inhibit COX1/2 activity in both LC cell lines with a higher preference toward COX2.

### 3.6. Effects of OC on HGF-Induced LC Cells Migration

**Figure 5:**
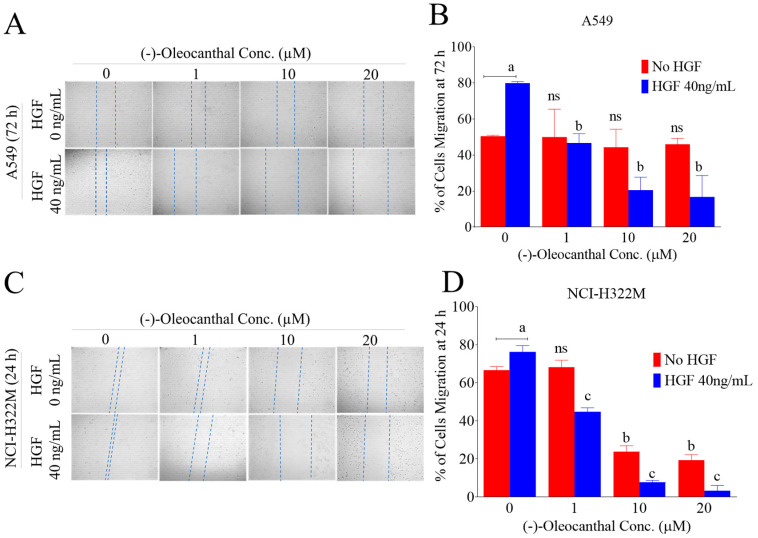
Antimigratory activity of OC treatments against LC cells. (**A**,**B**) Effects of OC treatment on the migration of A549 LC cells over 72 h treatment period with and without HGF 40 ng/mL treatments. (**C**,**D**) Effect of OC treatments on the migration of NCI-H322M LC cells over 24 h treatment period with and without HGF treatments. Right panel shows quantitative analysis of the percentage of gaps reduction (wound closures) in various treatment groups in A549 and NCI-H322M LC cells. Vertical bars indicate the percentage of wound closure of A549 at 72 h and NCI-H322M 24 h after wound scratching was calculated relative to the wound distance at time 0 (t**_0_**) ± SD in each treatment group. ^a^ *p* < 0.05 significantly different comparing no HGF with HGF 40 ng/mL controls. ^b^ *p* < 0.05 significantly different comparing OC treatments and vehicle-treated controls of no HGF. ^c^ *p* < 0.05 significantly different comparing OC treatments and vehicle-treated controls with the use of 40 ng/mL HGF. ns: Statically not significant.

Tumor cells migration is an important step forward to metastatic cascade. To test the effect of OC on LC cells migration, the wound-healing/scratch assay was used in presence and absence of mitogenic HGF using the human LC A549 and NCI-H322M cells (Figure 5). HGF at 40 ng/mL induced significant tumor cells migration leading to wound closure over 24 h treatment period for the NCI-H322M cells but it took 72 h for the A549 cells to close the wound. Therefore, OC treatment period was 24 h in NCI-H322M cells and 72 h in A549 cells. OC treatments significantly suppressed the HGF-induced cell migration in a dose-dependent manner in both cell lines (Figure 5A–D). OC treatments had minimal cellular migration inhibition against the A549 cells over 72 h in absence of HGF (Figure 5B). In NCI-H322M cells, OC treatments was much less potent in inhibiting the tumor cells migration in absence of HGF over 24 h treatment period as compared to its effects with the HGF treatment (Figure 5D). These results conclude that NCI-H322M is more migratory and c-MET-dependent and therefore more sensitive to OC treatments versus the A549 LC cells.

### 3.7. OC Treatment Activity against A549-Luc LC Metastasis in A Nude Mouse Tail Vein Model

**Figure 6:**
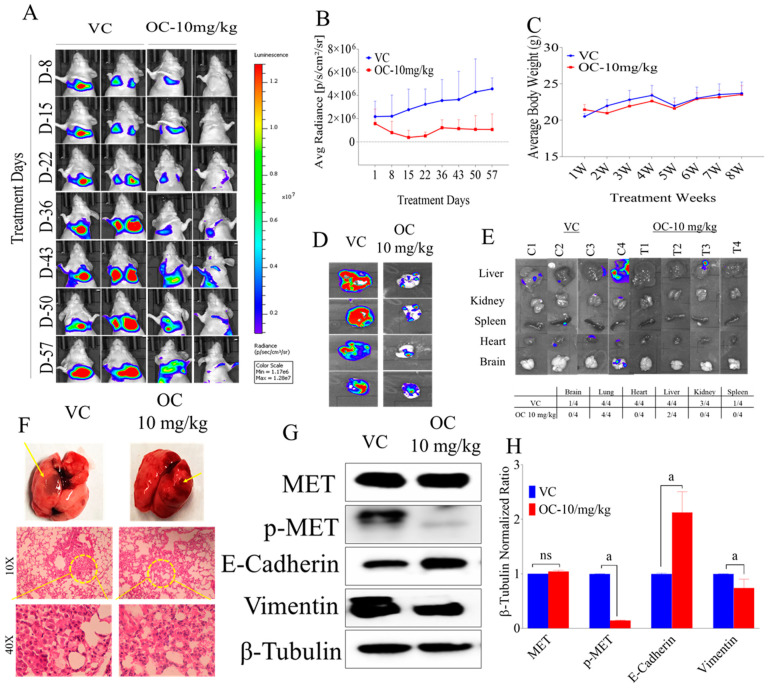
OC treatments suppressed the human LC A549-Luc progression and metastasis in a nude mouse tail vein model. (**A**) Weekly A549-Luc cells bioluminescence monitoring in intact animal. (**B**) Bioluminescence intensity monitoring in intact animals over the experiment course. (**C**) Average body weight monitoring of mice in different groups over the study period. (**D**) Bioluminescence imaging comparison of collected intact tumor-containing mouse lungs of OC-treated versus vehicle-treated groups. (**E**) Bioluminescence and morphological comparison of animal organs for OC-treated versus vehicle control-treated groups collected at the study end. (**F**) Representative H&E stained lung tissue of OC-treated versus vehicle control mouse at the experiment end. (**G**) Western blotting visualization of the effect of OC treatments on the total and activated c-MET, E-Cadherin and vimentin in the human A549-Luc lung tumor tissue lysates. (**H**) Scanning densitometry quantitation of total and activated c-MET, E-cadherin and vimentin in A549-Luc tumor cell lysates. Analysis was carried out in triplicates and the integrated optical density of each band was normalized with the corresponding density found for β-tubulin in the same blot. The vertical bars graph indicates the normalized integrated optical density of indicated marker bands visualized in each lane. ^a^ *p* < 0.05 significantly different compared to vehicle control. ns: Statically not significant.

Since OC treatments showed good in vitro antimigratory activities against A549 and NCI-H322M LC cell lines, the in vivo antimetastatic effect of OC treatments was examined in a nude mouse tail vein model. Mice injected with A549-Luc cells 5 × 10^6^ into the tail vein and immediately started daily oral 10 mg/kg OC treatments, which continued for 8 weeks. Weekly bioluminescence imaging was used to monitor the tumor progression and metastasis (Figure 6A). Bioluminescence imaging showed the persistent ability of OC treatments to effectively suppress the LC progression and metastasis over the study course (Figure 6A,B). Mice body weight was carefully monitored over the study course and showed no variations between OC and vehicle control treatments (Figure 6C). Bioluminescence imaging comparison of collected intact lungs of mice at the study end revealed potent suppression of LC progression in OC-treated group (Figure 6D). Bioluminescence imaging of other collected mice organs at the study end also proved OC treatments significant prevention of tumor metastasis to the brain, heart, kidney and spleen, in comparison with the vehicle control-treated animals (Figure 6E). Two out of four mice did not have any tumor metastasis in liver in OC-treated group unlike the vehicle control-treated mice, which showed four out of four tumor metastases in liver (Figure 6E). Morphological and histopathological examinations of mice lungs indicated the notable reduction of LC metastatic lesions in OC-treated group versus the vehicle control (Figure 6F). Western blotting analysis of collected LC tissue lysates showed potent suppression of c-MET activation, notable enhancement of the expression of the epithelial marker E-cadherin and downregulation of the mesenchymal marker vimentin in OC treatment group versus vehicle control- treated group (Figure 6G,H). These results clearly suggest the OC potential as a lead for the control of NSCLC.

### 3.8. Gene Signature of OC Treatments in Human A-549-Luc LC Tissues Using Human Clariom S Microarray Analysis

**Figure 7:**
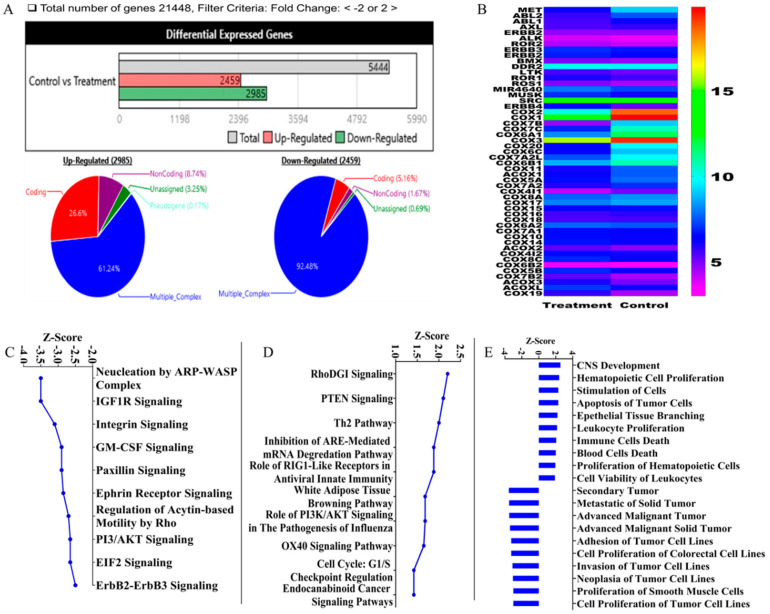
Microarray analysis of OC treatment effects on the non-small cell lung cancer (NSCLC) A549-Luc cell lysates using the human Clariom S array. Ingenuity Pathway Analysis (IPA) software used to analyze canonical pathways, functions and upstream targets, along with predicted activation scores. (**A**) Overview of genetic data and quantitative schematic representation of differentially expressed genes and interrelated gene expressions. (**B**) Representative differential expression of RTK and COX genes. (**C**) The topmost lowest z-score canonical pathways. (**D**) The topmost canonical pathways with highest z-score. (**E**) Predicted affected cellular functions with the highest and lowest z-scores.

The human microarray analysis was used to identify the OC treatments gene expression level signature at the A549-Luc LC tissues collected after animals sacrificed at the study end. The human Clariom S array serves as a next generation transcriptome-wide gene-level expression profiling tool, enabling the comprehensive coverage of all known well-annotated genes. A total of 5444 genes were affected (+/-) out of 21,448 genes (Figure 7A). Among them, 2985 genes upregulated and 2459 genes downregulated (Figure 7A). Among the upregulated genes, 61.2% gene originated from multiple complexes while 26.6% are coding genes. Among the downregulated genes, 92.5% genes originated from multiple complexes and only 5.2% are coding genes (Figure 7A). The hierarchical clustering showed the relative tyrosine kinase and COX genes expression in OC-treated tumor tissue versus those treated with vehicle control (Figure 7B). Extensive genes altered in response to OC treatments. Among the RTK family members affected by OC treatments, the c-MET was the topmost affected, with −7.72-fold downregulation versus the vehicle control (Table 1). Meanwhile in the COX genes family, COX2 was the most downregulated gene, with −332.4-fold change versus the vehicle control (Table 2).

Further, the Ingenuity Pathway Analysis (IPA) software was used to evaluate the pathways affected and to gain insights into gene-level signature of OC treatments in this NSCLC representative tumor. The z-score is an IPA metric, which translates overall biological function predicted activation or inhibition condition. Negative z-score means inhibited while positive scores implies activation. Several additional networks of pathways proved significantly affected by the OC treatments. The topmost 10 pathways with the lowest and highest z-values were predicted (Figure 7C,D, respectively). The activation score z indicates a pathway’s overall enhancement (positive values) or suppression (negative values) at different regulator levels. Examples of pathways negatively affected by OC treatments are nucleation by actin-related proteins- Wiskott–Aldrich syndrome protein (ARP-WASP) complex, insulin-like growth factor 1 (IGF-1), integrins, granulocyte-macrophage colony-stimulating factor (GM-CSF), paxillin, epherin receptor, Rho-driven acytin-based motility and PI3/Akt signaling pathways (Figure 7C). Examples of pathways positively affected by OC treatments are RhoDGI, phosphatase and tensin homolog (PTEN) and T helper type 2 (Th2) pathways (Figure 7D). In addition, IPA predicted strong negative z-scores to functions associated with secondary tumor formation, metastasis, invasion, adhesion, neoplasia and tumor cell proliferation, suggesting OC potential preventive effects (Figure 7E). Meanwhile IPA predicted strong positive z-scores to functions associated with central nervous system development, hematopoietic cells proliferation and apoptosis of tumor cells. Further, IPA analyzed a subset of downstream pathways, functions and regulators in the context of suppressing the upstream genes c-MET and COX2 following the OC treatments. The canonical pathway with the minimum z-score for c-MET was HGF signaling, while the maximum z-score pathway was FGF signaling. The corresponding minimum function was “Secondary tumor 3,” while the maximum function was “Development of central nervous system 2.” Overall, the IPA analysis indicated pathways and molecular mechanistic responses (either with positive or negative activation scores z) that clearly highlight the therapeutic potential of OC against NSCLC. A summarized IPA analysis predicted specific biological function responses that should be observed based on the detected gene expression level changes due to the OC treatment, with a z-score assigned to each function. Example of the functions with the highest z-score values include cell death and apoptosis (Figure 7E). IPA also provided a short list of predicted upstream targets, including RhoDGI and PTEN (Figure 7D).

**Figure 8:**
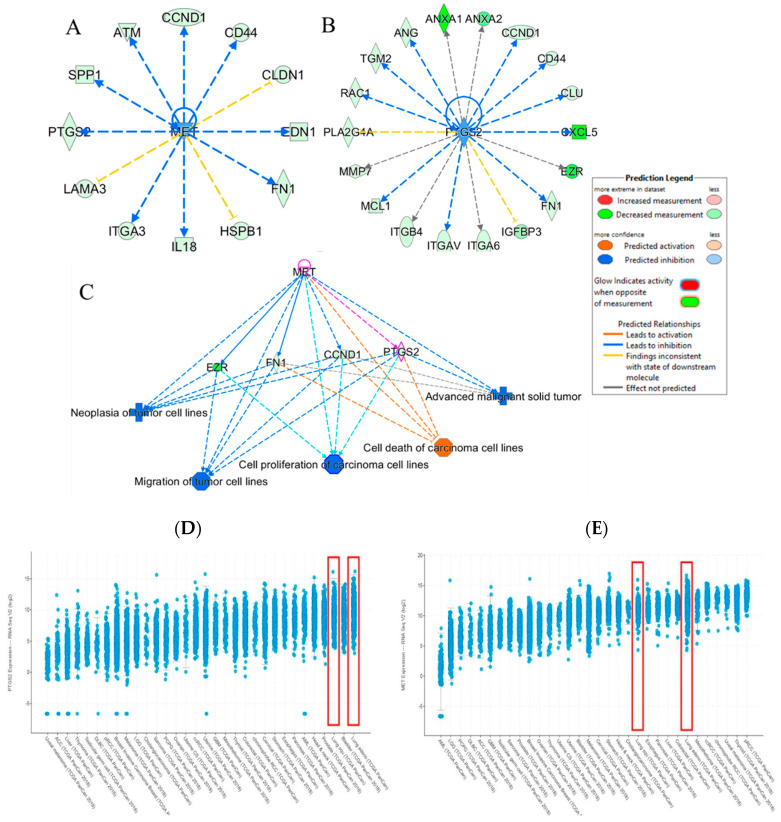
IPA-generated network mapping of OC treatment signal transduction effects through targeting c-MET and COX2 (PTGS2). (**A**) Predicted mapping of downstream pathways to be affected by OC treatment-induced c-MET modulation. (**B**) Predicted mapping of the downstream pathways to be affected by COX2 (PTGS2) suppression caused by OC treatment. (**C**) IPA-generated predicted outcomes and functions affected by the OC treatment dual suppression of c-MET and COX2 in NSCLC. The cBioportal for Cancer Genomics database (https://www.cbioportal.org/) indicates the predominance of the expression of the mRNAs of PTGS2 (**D**) and MET (**E**) genes in patient tumors of several lung adenocarcinoma and squamous cell lung carcinoma.

The IPA software was further used to analyze the most affected biological functions in tumor samples after OC treatment to better understand the biological relevance of the affected genes expression changes. Interestingly, most predicted suppressed categories included cell migration, proliferation, metastasis and induction of tumor cellular death-associated genes. Examples of regulated genes are cyclin D1 (CCND1), p53 apoptosis effector related to PMP22 (PERP), laminin subunit alpha 3 (LAMA3), interlukin 18 (IL18), fibronectin 1 (FN1), heat shock protein family B (small) member (HSPB1), claudin 1 (CLDN1) are expected to be downregulated in response to suppression of the upstream RTK c-MET expression (Figure 8A). The CCND1 is critical for tumor cell cycle, while laminins are essential for the formation and function of the basement membrane and regulation of cell migration and mechanical signal transduction. Fibronectin is also involved in tumor cell adhesion and migration, embryogenesis, wound healing, blood coagulation, host defense and metastasis. Meanwhile, the invasion and migration critical genes CCND1, ezrin (EZR), C-X-C motif chemokine ligand 5 (CXCL5), integrin α6 and β4 (ITGA6, ITGB4), matrix metalloprotease 7 (MMP7), clusterin (CLU), annexin A1 (ANXA1) were also downregulated in response to modulation of PTGS2 (COX2) expression by OC treatment (Figure 8B). Specifically EZR is a cytoplasmic peripheral membrane protein, which plays a key role in cell surface structure adhesion, migration and organization of various human cancers, including NSCLC. The CXCL5 is a member of the CXC chemokines subfamily, which promotes angiogenesis and remodelling of connective tissues and hence plays important role in cancer cell proliferation, migration and invasion. In addition, the ITGA6 and ITGB4 subunits may promote tumorigenesis and invassiveness. The main IPA analyses predicted outcomes due to combined downregulation of c-MET and PTGS2 by OC treatment in NSCLC include the suppression of tumor cell proliferation, migration and metastasis and induction of tumor cell death (Figure 8C). Exploration of the literature expression profile of c-MET and PTGS2 in clinical LC patients’ samples further highlighted their clinical significance. c-MET and PTGS2 proved highly expressed in several patient lung adenocarcinoma samples, along with several other tumor types in cBioPortal database (Figure 8D,E) [60,61].

## 4. Discussion

The EVOO-exclusive monophenolic *S*-(-)-oleocanthal has been documented as a valid c-MET kinase domain competitive inhibitory lead [41,42,43,44,45,46,47,48,49]. OC treatments suppressed the in vitro HGF-mediated proliferation and migration of the human LC cell lines A549 and NCI-H322M cells. This effect was less marked in absence of HGF. OC antiproliferative and antimigratory effects were also associated with the suppression of activated c-MET in treated LC cell lines. OC treatments minimally affected the viability of the non-tumorigenic human microvascular endothelial cells HMVEC at a concentration two-fold higher than its LC suppressive concentration, suggesting its potential selectivity to LC cells. Daily 10 mg/kg oral OC treatments noticeably resulted in a significant inhibition of the progression and metastasis of the NSCLC A549-Luc cells in a nude mouse tail vein model compared to the vehicle-treated control animals. This in vivo LC suppressive effect was also associated with significant inhibition of the c-MET activation in tumor cell lysates collected from treated animals at the experiment end as compared to the vehicle control group.

The c-MET RTK is a potential oncogenic driver in NSCLC cell lines [16,18,30]. Accumulating mounting evidences supported the role of HGF and its receptor c-MET in the human LC development, progression and developing resistance to targeted therapies [12,16,18,62]. Dysregulation of c-MET signaling–mediated proliferation, migration, invasion and angiogenesis through overexpression of MET and amplification or mutation of the MET gene has been widely demonstrated in oncogenic processes across multiple tumor types [11,42]. Moreover, it is notable that all mechanisms of c-MET dysregulation have been documented in NSCLC [11]. Responses to c-MET tyrosine kinase inhibitors have been documented in clinical trials in patients with c-MET-amplified or c-MET-overexpressing NSCLC [11]. Earlier studies validated OC ability to suppress breast cancer progression via ATP-competitive inhibition of the c-MET kinase domain [42,44,46]. This study reports for the first time the OC activity against LC growth and migration in vitro and progression and metastasis in vivo through blocking the c-MET kinase activation.

Several c-MET-targeted agents have been developed and used in clinical experiments but still unable to get the FDA approval due to disappointing clinical trial results [16,63]. c-MET phosphorylation at Y1234/1235 and Y1349 could be detected in the NSCLC tumor samples but there was no significant correlation between c-MET expression and activation. Recently, a study proved c-MET could be regulated by COX2 and could phosphorylate and activate the T-lymphokine-activated killer cell-originated protein kinase (TOPK) at its Y74 site [63]. On the other hand, consistent exposure of NSCLC cell cultures to HGF treatments reproducibly induced COX-2 expression and high cell proliferation rates [30,31]. COX2 proved one of the downstream effectors of activated c-MET signaling in response to HGF induction [30,31]. COX2 is the inducible rate-limiting enzyme responsible for the prostaglandin E2 (PGE_2_) production. PGE_2_ mediates pro-tumor effects through paracrine stimulation of inflammatory cells and autocrine stimulation of its receptors in tumor cells [28,30,64]. Induction of COX2 in NSCLC occurred through both the extracellular signal-regulated kinase 1/2 (ERK1/2) and p38 pathways [30]. COX2 has been already proved a critical clinical marker for poor prognosis in lung, colorectal, gastric, head and neck cancers [24,25,26,27,28,29]. The serum level of COX2 was also validated as a biomarker for EGFR mutation, response for EGFR TKIs, and progression-free survival in LC patients [65]. Meanwhile, inhibition of COX-2 limits the production of prostaglandins, which are known to stimulate cell proliferation, induce invasiveness and mediate angiogenesis [30]. Targeting COX2 proved acceptable strategy to control lung and other malignancies [28,29,30,31,32]. Earlier in 2005, Beauchamp et al. identified OC as the non-steroidal anti-inflammatory drug (NSAID) ibuprofen-like active ingredient in EVOO [40]. Meanwhile, ibuprofen and other NSAIDs showed documented anticancer effects through the suppression of various COX isoforms [40]. OC treatment significantly reduced the activity of COX1 and COX2 in LC cells in vitro. This study also assessed the in vivo effects of the ibuprofen-like OC on the COX1 and COX2 expression levels in the human NSCLC A549 cells via the microarray analysis. OC treatments showed higher selectively toward COX2 versus COX1 and COX3 expressions. The COX2 inhibitory effect of OC in LC cells can be in-part a downstream effect for the c-MET inhibition. The dual dysregulation of c-MET and COX2 reported to significantly enhance LC proliferation, survival, invasiveness, metastasis and resistance [31]. Thus, co-targeting COX2 and c-MET proved effective strategy to suppress LC tumorigenesis and reverse its resistance [31]. Investigation of LC patient biopsy samples showed increased c-MET expression in the primary tumor and correlated its level with a higher risk of metastases [66]. This study showed the ability of daily oral OC 10 mg/kg dosing to prevent the metastasis of A549-Luc LC cells to brain, heart, kidney and spleen in a nude mouse tail vein model. In addition, OC treatment notably suppressed the A549-Luc LC cells metastasis to animal livers versus the vehicle control group. Western blot analysis of collected animal tumor samples confirmed the significant inhibition of activated c-MET kinase in OC treated group versus vehicle control.

The first step in metastatic cascade is the Epithelial-to-Mesenchymal Transition (EMT) [42]. Epithelial cells lose cell-cell contacts, apical-basal polarity and acquire mesenchymal phenotype in EMT [42]. EMT is involved in cancer progression, particularly during invasion, intravasation and migration. E-cadherins are a family of transmembrane glycoproteins that mediate cell-cell adhesion and promote cells polarity. The loss of E-cadherin disrupts cells adhesion and polarity, enabling tumor cells metastasis. Meanwhile vimentin is an intermediate filament protein normally expressed in mesenchymal cells and it regulates cellular migration [42]. This study showed the significant in vitro anti-migratory activity of OC treatments in two LC cell lines. OC treatment also showed antimetastatic activity in vivo, which was associated with significant increase of E-cadherin and remarkable decrease of vimentin expressions in collected tumor sample lysates at the study end. These results mechanistically highlight the lead candidacy and improved efficacy of OC against LC cancer metastasis.

The transcriptome array results strongly indicate that OC treatments modulated the NSCLC A549 cells progression and metastasis through significantly suppressing c-MET and COX2 activities and their downstream pathway genes. Microarray results proved the ability of OC to significantly reduce the total c-MET expression level in A549 LC tissues by 7.7-fold versus the vehicle control. This inhibition was 2.2-fold higher than the nearest RTK family member ABL2 (Table 1). In addition, OC reduced the COX2 expression in A549 tumor tissues by 332.4-fold versus the vehicle control (Table 2). OC treatment also reduced the COX1 expression by 132.3-fold. This translates to 2.5-fold selectivity for OC to COX2 versus COX1. OC also reduced the COX3 expression in A549 tumor tissues by 12-fold versus the vehicle control. This also translates into 27.7-fold OC selectivity to COX2 versus COX3, suggesting a good degree of OC treatment selectivity to COX2 and qualifying OC as a dual c-MET/COX2 inhibitor. Although OC reduced the total c-MET expression level by 7.7-fold in A549-Luc tumor tissues, OC acts mainly as a competitive c-MET kinase inhibitor [42,44]. This can justify the significant suppression of several c-MET downstream effectors, including COX2 and several others. Beside the c-MET and COX2 suppression, the microarray results also showed that OC treatment downregulated over 50% of CEACAM6, AKRC1, AKR1C3 and AKR1B10 expression in the A549 NSCLC tissues versus vehicle control-treated group. Literature already proved that the expression of the signaling molecules carcinoembryonic antigen-related cell adhesion molecule 6 (CEACAM6) and cystatin SN (CST1) regulate the LC migration while CEACAM1 regulates LC adhesion [67]. The aldo-keto reductase family 1, member B10 (aldose reductase, AKR1B10) overexpression proved a valid prognostic factor for high recurrence risk in patients with resected LC [68]. AKRC1 and AKR1C3 are regulated by nuclear factor-Y and found to be linked to LC in heavy smokers [69].

Based on the microarray data, IPA analysis further identified several pathways that had low activation scores and directly related to LC proliferation, migration, invasion, metastasis and cells movement (Figure 7C,D). Indicatively, the actin nucleation by ARP-WASP Complex pathway, with the lowest activation score, is associated with motility and several other biological functions [70]. The cytoskeleton actin is a dynamic filament network that is essential for cell movement during embryonic development, polarization, morphogenesis, cell division, immune system function and tumor cells metastasis. Members of the WASP family such as N-WASP and at least three variants of SCAR/WAVE (WAVE1-3) coordinate with CDC42 and PIP2 and play a central role in regulating actin-based cell motility [70]. Furthermore, IPA predicted strong negative z-scores for the insulin-like growth factor (IGF) and integrin signaling pathway (Figure 7C,D). Clinical studies have shown that increased IGF-1R activity is implicated in activated cancer cells proliferation, migration and invasion [71]. In addition, increased serum levels of IGF-1 have been observed in cancers of the lung, colon, prostate and breast [71]. The integrins are a superfamily of heterodimeric transmembrane receptors responsible for the cellular adhesion to extracellular matrix (ECM) proteins, which further mediate signals for the control of diverse cellular functions, including survival, proliferation, differentiation, adhesion and migration [71]. The predicted negative scores for PI3/AKT and paxillin signaling pathways correlate well with literature-validated OC anti-invasive activity through downregulating PI3/AKT and paxillin signaling pathways in breast cancer [72]. Meanwhile, IPA further identified the RhoDGI and PTEN signaling as the pathways with the highest activation scores. PTEN is recognized as a negative regulator for the PI3-kinase/Akt signaling pathway, which controls the cell cycle progression and cell death [73]. OC treatment also predicted to induce several genes associated with promoting cell death, cell-cell adhesion, inducing apoptosis and limiting metastasis. Studies showed that expression of CXCL5 was significantly higher in LC and associated with high chemokine (C-X-C motif) receptor 2 expression leading to poor differentiation [74]. Recently, ezrin (EZR) proved to promote breast cancer progression by modulating the Akt signaling [75]. Meanwhile, fibronectin-mediated activation of focal adhesion kinase (FAK) can lead to activated LC metastasis through ERK or PI3K/Akt regulation of MMP9/calpain-2 or MMP9/RhoA activity, respectively [76]. The cyclin D1 is a key abnormality in LC [77]. Interestingly, IPA analysis of microarray data of OC-treated A549 LC lysates revealed that CXCL5, EZR, FN1 and CCND1 have been downregulated through the modulating effect of OC treatment on c-MET and COX2 (PTGS2), which further result-in more effective suppression of LC proliferation and migration. Overall, the microarray data further validated OC as a viable lead capable to suppress the LC progression and metastasis via the suppression of c-MET and its downstream effector COX2.

## 5. Conclusions

*S*-(-)-Oleocanthal, an exclusive olive-oil phenolic component, has captured an increasing interest as a potential anticancer lead. Collectively, the present study findings validate OC as a potential entity appropriate for use to control LC through its dual c-MET and COX2 modulation. Microarray data further confirmed the positive OC therapeutic potential against LC at the gene levels, validating its c-MET/COX2 suppressive effects, along with the suppression of downstream network of genes associated with LC proliferation, migration and metastasis.

## Figures and Tables

**Table 1:** A representative list of the affected RTK genes showing differential fold-expression change in OC-treated as compared to vehicle control groups.

Gene	Fold Change	Description
MET	−7.72	hepatocyte growth factor receptor
ABL2	−3.47	Abelson murine leukemia viral oncogene homolog 2
ABL1	−1.53	Abelson murine leukemia viral oncogene homolog 1
AXL	−1.21	AXL receptor tyrosine kinase
ERBB2	−1.01	erb-b2 receptor tyrosine kinase 2
ALK	1.20	anaplastic lymphoma receptor tyrosine kinase
ROR2	1.22	receptor tyrosine kinase-like orphan receptor 2
ERBB3	1.22	erb-b2 receptor tyrosine kinase 3
MIR4728; ERBB2	1.22	microRNA 4728; erb-b2 receptor tyrosine kinase 2
BMX	1.23	BMX non-receptor tyrosine kinase
DDR2	1.27	discoidin domain receptor tyrosine kinase 2
LTK	1.32	leukocyte receptor tyrosine kinase
ROR1	1.32	receptor tyrosine kinase-like orphan receptor 1
ROS1	1.35	ROS proto-oncogene 1, receptor tyrosine kinase
DDR1; MIR4640	1.61	discoidin domain receptor tyrosine kinase 1; microRNA 4640
MUSK	1.93	muscle, skeletal, receptor tyrosine kinase
SRC	1.95	SRC proto-oncogene, non-receptor tyrosine kinase
ERBB4	2.04	Erb-b2 receptor tyrosine kinase 4

**Table 2:** A representative list of COX genes showing differential fold-expression changes in OC- treated group as compared to vehicle control group.

Gene	Fold Change	Description
COX2	−332.39	cytochrome c oxidase subunit II
COX1	−132.32	cytochrome c oxidase subunit I
COX7B	−21.62	cytochrome c oxidase subunit VIIb
COX7C	−13.52	cytochrome c oxidase subunit VIIc; microRNA 3607
COX6A1	−12.08	cytochrome c oxidase subunit VIa polypeptide 1
COX3	−12.00	ATP synthase F0 subunit 8; ATP synthase F0 subunit 6; cytochrome c oxidase III
COX20	−10.32	cytochrome c oxidase assembly factor
COX6C	−6.34	cytochrome c oxidase subunit VIc
COX7A2L	−4.87	cytochrome c oxidase subunit VIIa polypeptide 2 like
COX6B1	−4.18	cytochrome c oxidase subunit VIb polypeptide 1 (ubiquitous)
COX11	−2.85	cytochrome c oxidase copper chaperone
COX5A	−2.27	cytochrome c oxidase subunit Va
COX7A2	−2.10	cytochrome c oxidase subunit VIIa polypeptide 2 (liver)
COX4I1	−1.79	cytochrome c oxidase subunit IV isoform 1
COX8A	−1.53	cytochrome c oxidase subunit VIIIA (ubiquitous)
COX17	−1.41	cytochrome c oxidase copper chaperone
COX15	−1.25	cytochrome c oxidase assembly homolog 15 (yeast)
COX16	−1.19	cytochrome c oxidase assembly homolog
COX18	−1.06	cytochrome c oxidase assembly factor
COX6A2	1.14	cytochrome c oxidase subunit VIa polypeptide 2
COX7A1	1.16	cytochrome c oxidase subunit VIIa polypeptide 1 (muscle)
COX10	1.24	heme A: farnesyltransferase cytochrome c oxidase assembly factor
COX14	1.37	cytochrome c oxidase assembly factor
COX4I2	1.4	cytochrome c oxidase subunit IV isoform 2 (lung)
COX8C	1.52	cytochrome c oxidase subunit VIIIC
COX6B2	1.58	cytochrome c oxidase subunit VIb polypeptide 2 (testis)
COX5B	1.59	cytochrome c oxidase subunit Vb
COX7B2	1.86	cytochrome c oxidase subunit VIIb2
COX19	2.56	cytochrome c oxidase assembly factor
